# Improvement in the Tensile Bond Strength between 3Y-TZP Ceramic and Enamel by Surface Treatments

**DOI:** 10.3390/ma9080702

**Published:** 2016-08-18

**Authors:** Seon-Mi Byeon, Yong-Seok Jang, Min-Ho Lee, Tae-Sung Bae

**Affiliations:** Department of Dental Biomaterials and Institute of Biodegradable Material, Institute of Oral Bioscience and BK21 plus project, School of Dentistry, Chonbuk National University 664-14, 567 Baekje-daero, deokjin-gu, Jeonju-si, Jeollabuk-do 54896, Korea; seonmi793@gmail.com (S.-M.B.); Yjang@jbnu.ac.kr (Y.-S.J.); mh@jbnu.ac.kr (M.-H.L.)

**Keywords:** yttria-stabilized tetragonal zirconia polycrystal (Y-TZP), tensile bond strength, alumina sandblasting, selective infiltration etching (SIE), MDP-containing silane primer

## Abstract

This study examined the effects of 3 mol % yttria-stabilized tetragonal zirconia polycrystal (3Y-TZP) ceramic surface treatments on the tensile bond strength and surface characteristics of enamel. To measure the tensile bond strength, the 3Y-TZP and tooth specimens were manufactured in a mini-dumbbell shape and divided into four groups based on the type of 3Y-TZP surface treatment: polishing (P), 110 µm alumina sandblasting (S), 110 µm alumina sandblasting combined with selective infiltration etching (SS), and 110 µm alumina sandblasting combined with MDP (10-methacryloyloxydecyl dihydrogen phosphate)-containing silane primer (SP). After surface treatment, the surface roughness, wettability, and surface changes were examined, and the tensile bond strength was measured. The mean values (from lowest to highest) for tensile bond strength (MPa) were as follows: P, 8.94 ± 2.30; S, 21.33 ± 2.00; SS, 26.67 ± 4.76; and SP, 31.74 ± 2.66. Compared to the P group, the mean surface roughness was significantly increased, and the mean contact angle was significantly decreased, while wettability was increased in the other groups. Therefore, surface treatment with 110 µm alumina sandblasting and MDP-containing silane primer is suitable for clinical applications, as it considerably improves the bond strength between 3Y-TZP and enamel.

## 1. Introduction

The yttria-stabilized polycrystalline tetragonal zirconia (Y-TZP) ceramic is used widely in the clinic for esthetic restorations, because of its excellent esthetic performance and biocompatibility, as well as its high strength and fracture toughness, all of which demonstrate its superiority to other all-ceramic materials [1]. Zirconia is also used for partial fixed dental prostheses (PFDP), monolithic crowns in the anterior and posterior regions, and all-ceramic crowns [2].

For the long-term success of esthetic restorations using zirconia, it is essential to enhance retention and resistance of the restoration through strong bonding with the tooth structure. The methods generally used to achieve this outcome include the following: (i) expanding the surface area of the ceramic to enhance the bond strength with the tooth structure; (ii) hydrofluoric acid etching to create a stronger micro-mechanical interlock; and (iii) the use of silane primer to achieve chemical bond strength [3]. However, hydrofluoric acid etching cannot be performed on zirconia because it does not contain glass, and silane primer cannot provide sufficient bond strength by itself as no reaction occurs because of the lack of silica in zirconia means that no reaction occurs [4]. Therefore, surface treatments of zirconia have been explored to overcome these limitations using methods such as grinding/polishing, sandblasting, surface coating, laser treatment, and primer treatment [1,5].

Sandblasting with aluminum oxide particles of different sizes produces irregularities and shallow pits in the zirconia surface, which enhances the micro-mechanical retention [6,7]. In addition, the surface of zirconia that has been coated with a 10-methacryloyloxydecyl dihydrogen phosphate (MDP)-containing silane primer after sandblasting is more effective at providing chemical and micro-mechanical bond strength with the tooth structure [8]. Silane or MDP-containing silane primer treatments improve the chemical bond strength of zirconia [9]. In particular, MDP-containing silane primers contain a methacryloyl group for copolymerization, a hydrophobic decyl group that is unaffected by moisture of the dental pulp fluid or the saliva, and a hydrophilic dihydrogen phosphate group for chemical bonding with hydroxyapatite and metal oxides [10]. 

The selective infiltration etching (SIE) technique is effective in increasing the bond strength between zirconia and resin cements. After heating a glass infiltration agent, it is allowed to penetrate into the void space between the zirconia grains and causes them to become re-arranged. Once the glass infiltration agent has been removed using hydrofluoric acid, the zirconia develops inter-grain nano-porosity, generating a relatively rough and reactive surface that exhibits improved micro-mechanical bond strength with resin cements [11].

Typically, when measuring the bond strength between ceramics and the tooth structure, shear and tensile bond strength tests have been used according to ISO/TS 11405. The shear bond strength test is relatively simple and is the most commonly used method for measuring the bond strength between two materials. However, caution is required because stress distribution is uneven, which may lead to a large standard deviation. By comparison, the tensile bond strength test is more accurate, and it enables the failure modes to be observed more clearly after the experiment. One drawback of the tensile bond strength test is that it is more technically difficult than the shear bond strength test; this is because an imprecise loading point or bonded surface between the two materials can lead to inaccurate measurements of bond strength [12,13,14]. Nevertheless, the bonded surface is accurately controlled to a narrow area of approximately 1 mm^2^, which enables even distribution of stress at the interface and increases the bond strength [15]. Therefore, to obtain more accurate measurements of the bond strength between zirconia and the tooth, this study measured tensile bond strength across a bonded surface area of approximately 1 mm^2^. From a technical standpoint, particular efforts were made to reduce the error in the shape of the specimens used in the experiment and to apply the load precisely parallel to the jig that was holding each specimen.

This study evaluated the bond strength between 3Y-TZP (3 mol % yttria-stabilized tetragonal zirconia polycrystal) ceramic and enamel after applying sandblasting with various surface treatment methods, including the SIE technique, and the use of MDP-containing silane primer. In addition, tensile bond strength and failure modes were assessed and surface characteristics were examined. 

## 2. Materials and Methods

### 2.1. Preparation of Specimens and Surface Treatments

**Figure 1:**
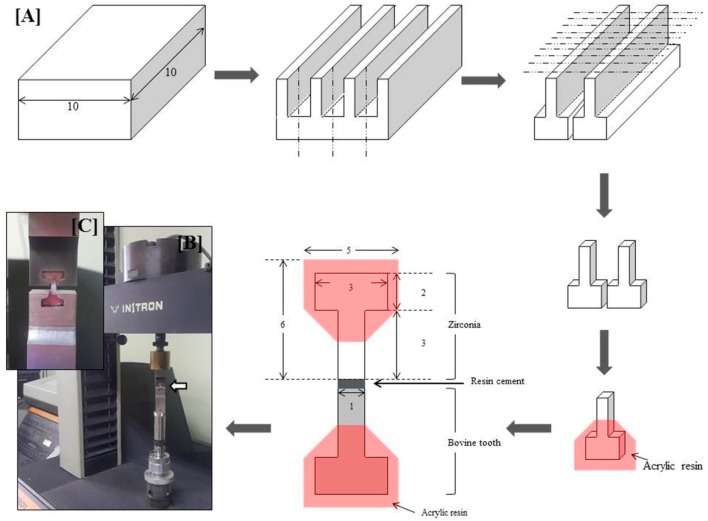
(**A**) Schematic illustration of mini-dumbbell specimen preparation for the tensile bond strength test (unit: mm); (**B**) Tensile bond strength testing machine and jig; (**C**) Magnified image of the specimen held by the jig (indicated by white arrow in (**B**)).

The experimental materials included a pre-sintered 3 mol % yttria-stabilized tetragonal zirconia polycrystal (3Y-TZP) ceramic block (Zirmon, Kuwotech, Gwangju, Korea) and 50 non-carious bovine incisors. The procedure involved cutting the 3Y-TZP and bovine teeth in distilled water using a high-speed diamond saw (Isomet 5000, Buehler, Lake Bluff, IL, USA) according to the procedure illustrated in Figure 1A. The ceramic (3Y-TZP) specimens were sintered according to manufacturer’s instructions (final bonding surface area = 1 mm^2^). Subsequently, both 3Y-TZP and enamel specimens were embedded separately in acrylic resin using mini-dumbbell molds and were fabricated to fit the tensile testing jig (Figure 1A). The bonding surface of 3Y-TZP and teeth specimens was polished with #400–1200 SiC sandpaper and then the specimens were washed for 5 min using distilled water with an ultrasonic cleaner, and were dried. For sandblasting, the bonding surface of 3Y-TZP was sandblasted with 110 µm alumina (Al_2_O_3_) particles from a distance of 10 mm for 10 s, at 0.4 MPa. The sandblasted specimens were heated at 900 °C for 10 min in order to stabilize the monoclinic phase to the tetragonal phase. Thereafter, the sandblasted specimens were either treated with selective infiltration etching (SIE), or an MDP-containing silane primer was applied, depending on the group. The details are displayed in Table 1.

After treatments of the 3Y-TZP specimens (Table 1), the surface of the enamel was etched for 15 s with 32% phosphoric acid gel (Scotchbond Universal Etchant, 3M ESPE, St. Paul, MN, USA) and washed for 20 s to remove the remaining phosphoric acid gel. A bonding agent (Single Bond Universal Adhesive, 3M ESPE) was applied to the surface of both the enamel and the 3Y-TZP specimens for 20 s. Resin cement (Rely X Ultimate, 3M ESPE) was then applied to the surface of the 3Y-TZP specimen, and the enamel was placed on the resin cement. Excess resin cement was removed with an explorer, and the remaining resin cement was light-cured for 10 s on each side at an angle of 45°.

### 2.2. Surface Roughness, Wettability, and Morphology

To compare the relationship between surface roughness and wettability [16] of the 3Y-TZP specimens after the surface treatments, the specimens were analyzed using a surface roughness tester (SV-3000, Mitutoyo, Tokyo, Japan) (one per group) and a surface electro optics instrument (PHX-300 Touch, Surface Electro Optics, Suwon, Korea) (3 per group). The surface roughness tester was used with the diamond stylus to move along a 5 mm length at a speed of 0.2 mm/s, and the surface electro optics instrument was used by the sessile drop method on the specimen surface after placing a droplet of distilled water for 5 s. The contact angle was analyzed using the Sufaceware8 software (Surface Electro Optics, Suwon, Korea).

The morphology of the specimens after the surface treatments was observed by field emission scanning electron microscopy (FE-SEM; SU-70, Hitachi, Tokyo, Japan) (1 per group) and atomic force microscopy (AFM; Multimade-8, Bruker, Billerica, MA, USA) (1 per group). 

### 2.3. Tensile Bond Strength (TBS) Test and Failure Modes

Fifty of the specimens of enamel that had been bonded to 3Y-TZP were stored in distilled water at 37 ± 1 °C for 24 h (the first test in the ISO/TS 11405 standards [17]; short-term storage). A universal testing machine (Model 4201, Instron, Canton, MA, USA) was then used to measure the tensile bond strength (TBS) between the 3Y-TZP and the enamel. The bonded specimens were fixed to the jig having a mini-dumbbell shape, and a tensile force was loaded at a crosshead speed of 1.0 mm/min (Figure 1B,C).

After measuring TBS, the bonded surfaces were analyzed using a light microscope (DM 2500M, Leica Microsystems, Wetzlar, Germany) at ×20 magnification to examine the failure modes between the 3Y-TZP and the enamel. Failure modes were classified into adhesive failure, cohesive failure, or mixed failure, and the ratios of each were calculated.

### 2.4. Statistical Analysis

Statistical processing was performed using SPSS 12.0 software (SPSS, Chicago, IL, USA). After the experiment, a one-way ANOVA (ANalysis Of VAriance) test was performed, and variables showing significant differences were evaluated by Tukey’s combined comparison test (*p*-value = 0.05).

## 3. Results

**Figure 2:**
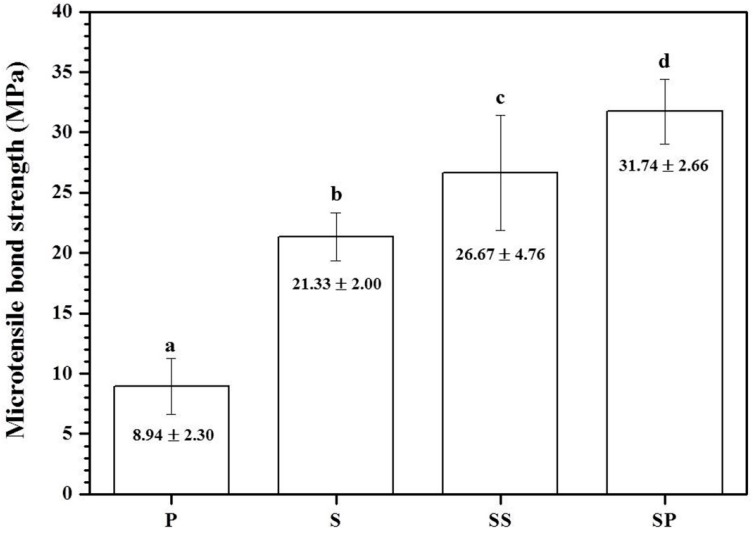
Tensile bond strength (TBS) of enamel bonded to 3Y-TZP after surface treatments. Bars indicate the standard deviation. P: Polishing; S: Sandblasting; SS: Sandblasting + SIE technique; SP: Sandblasting + MDP-containing silane primer. a–d: Groups shown with different letters were significantly different (*p* < 0.05).

**Figure 3:**
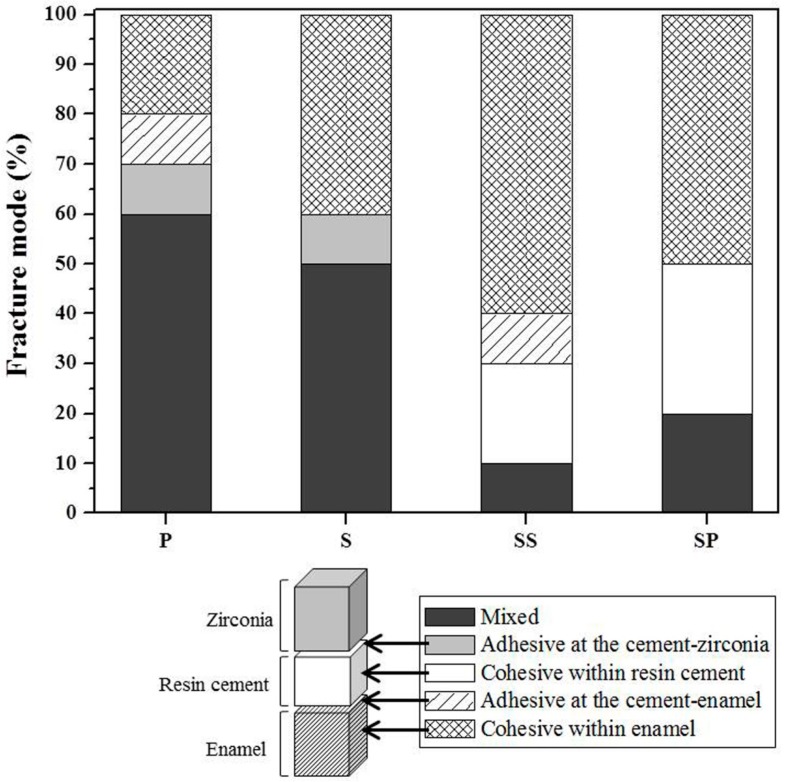
Percentages (%) of the fracture mode after tensile bond strength test of enamel bonded to 3Y-TZP. P: Polishing; S: Sandblasting; SS: Sandblasting + SIE technique; SP: Sandblasting + MDP-containing silane primer.

Figure 2 shows the mean and standard deviation of TBS for each group, and Figure 3 shows the failure modes at the bonded surface between the 3Y-TZP and the enamel. The P group showed the lowest TBS value, while the failure modes were 80% mixed and adhesive failure, and 20% cohesive failure. After surface treatment, TBS values showed a significant increase in all groups (S, SS, and SP) relative to the P group (*p* < 0.05). The failure modes of the S group were 60% mixed and adhesive failure, and 40% cohesive failure. The SS group had a significantly higher TBS value than the S group (*p* < 0.05). Meanwhile, the failure modes of the SS group were 20% mixed and adhesive failure, and 80% cohesive failure. The SP group showed the highest TBS value, with failure modes of 20% mixed failure and 80% cohesive failure.

**Figure 4:**
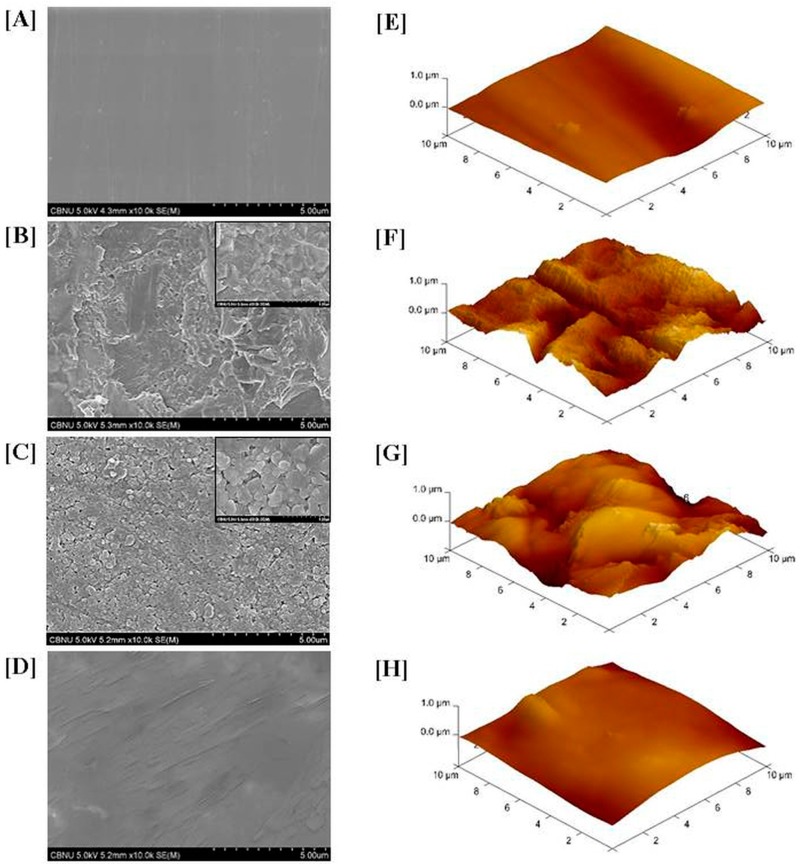
FE-SEM images (magnifications: ×10,000, ×50,000) and AFM images (spot size: 10 μm × 10 μm). (**A**,**E**) Polishing; (**B**,**F**) Sandblasting; (**C**,**G**) Sandblasting + SIE technique; (**D**,**H**) Sandblasting + MDP-containing silane primer.

**Figure 5:**
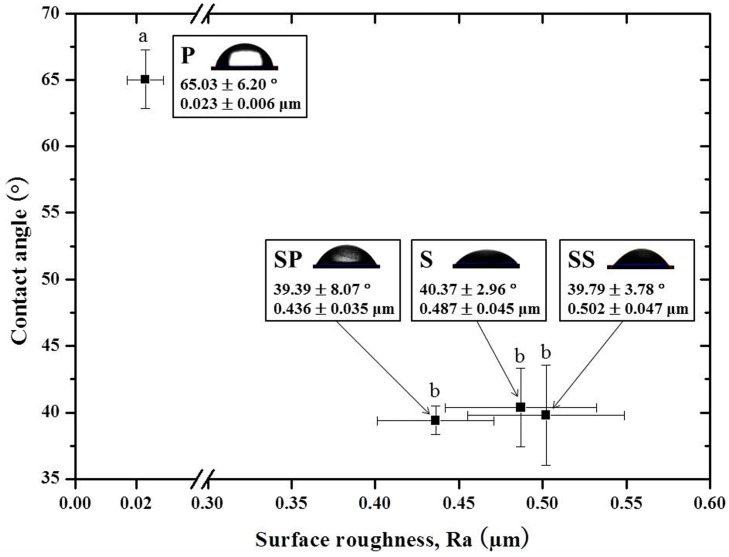
Contact angle as a function of surface roughness values after 3Y-TZP surface treatments. Bars indicate the standard deviation. P: Polishing; S: Sandblasting; SS: Sandblasting + SIE technique; SP: Sandblasting + MDP-containing silane primer. a, b: Groups shown with different letters were significantly different (*p* < 0.05).

Compared to the P group (Figure 4A,E), all treated groups (S, SS, and SP) showed a significant increase in the surface roughness value, a significant decrease in the contact angle, and an increase in wettability (*p* < 0.05, Figure 5). In the S group, the roughened surface was generated by 110 µm A1_2_O_3_ sandblasting (Figure 4B,F). The SS group showed the highest surface roughness value (Figure 5), whereas the SIE technique resulted in the formation of a porous surface (Figure 4C,G). In the SP group, application of the MDP-containing silane primer produced a layer of coating that resulted in a relatively smooth surface (Figure 4D,H) showing a significantly reduced surface roughness value compared with those in the other surface treatment groups (*p* < 0.05).

## 4. Discussion

The most important factor in enhancing the durability of ceramic restorations is chemical and mechanical bonding to the tooth structure. Moreover, determining the optimal method for improving bond strength between restorative dental materials and teeth is essential for providing patients with a positive esthetic outcome, and for ensuring high long-term success rates. Unlike glass ceramics, zirconia cannot be treated by hydrofluoric acid etching because it does not contain glass. Furthermore, chemical bonding via silanization is also impractical, due to the lack of silica in zirconia [18]. This means that more effective surface treatment methods need to be explored to increase bond strength between zirconia and the tooth structure.

Therefore, this study investigated various methods for the surface treatment of zirconia after 110 µm Al_2_O_3_ sandblasting, including the SIE technique, and application of an MDP-containing silane primer. These surface-treated zirconia specimens were then evaluated for their tensile bond strength with enamel according to the TBS test, and the surface characteristics of treated zirconia were examined to determine the factors that comprise effective bonding. Previous analysis of bond strength [19] revealed that the surface of the substrate needs to be clean in order to achieve effective adhesion; it also needs to have high wettability in order to make close contact with the adhesives, and it should be capable of forming chemical or micro-mechanical adhesion.

Sandblasting was used in all treatment groups because it helps to increase bond strength by removing contaminants on the surface of the zirconia. Sandblasting also increases surface roughness as well as surface area and surface energy, thus making it possible to achieve stronger mechanical durability of the restoration. Moreover, it can also increase wettability of resin cements or silane primers [20,21].

In the current study, the S group showed a significantly greater increase in the TBS value than the P group, from 8.94 ± 2.30 to 21.33 ± 2.00 MPa. Inspection of failure modes after measuring bond strength showed an increase in the rate of cohesive failure of the enamel from 20% (P group) to 40% (S group). Zandparsa et al. [22] proposed that failure modes can be divided into adhesive failure and cohesive failure, and further noted that a weak bond strength is associated with a higher rate of adhesive failure between the resin cement and the zirconia, or between the resin cement and the enamel. Conversely, the bond strength that is similar to the strength of the resin cement showed a relative increase in cohesive failure within the enamel or the resin cement rather than adhesive failure. In addition, analysis of changes in the sandblasted zirconia surface for the S group showed overall roughness and shallow pits (Figure 4B,F) and a significant increase in surface roughness and wettability (Figure 5).

The SIE technique facilitates micro-mechanical bond strength with the resin cement by forming inter-grain nano-porosity in the zirconia surface [11,14]. The TBS value of the SS group was increased compared to that of the S group. In addition, the rate of cohesive failure increased considerably to 80%, and the porous zirconia surface showed the formation of undercuts (Figure 4C) and an increase in roughness and wettability.

As zirconia does not contain silica, the use of a silane primer alone does not help to increase bonding with the resin cement [23]. Therefore, before applying the MDP-containing silane primer, sandblasting was performed on the zirconia to enhance mechanical adhesion [24]. The SP group showed the highest bond strength, at 31.74 ± 2.66 MPa, representing a significant increase compared to the other groups (P, S, or SS). Moreover, the ratio of failure modes was 20% mixed failure (adhesive failure and cohesive failure) and 80% cohesive failure. The contact angle was significantly decreased in the SP, which also showed the highest wettability. This is consistent with studies showing that when an MDP-containing silane primer is applied after sandblasting, there is an increase in surface roughness; this is because the primer binds directly to hydroxyl groups on the zirconia surface and enhances the stability against hydrolysis, which in turn improves bond strength with an adhesive [25]. Furthermore, Blatz et al. [26] and Fukegawa et al. [10] have reported that the dihydrogen phosphate group in MDP (10-methacryloyloxydecyl dihydrogen phosphate) forms strong chemical interactions with hydroxyapatite crystals in the enamel and zirconium oxides in the zirconia surface. Thus, application of MDP-containing silane primer after sandblasting can promote strong bond strength with the enamel, thus enhancing the durability and long-term success of zirconia-based restorative outcomes.

Based on the results of this study, the rate of cohesive failure increased in the SS and SP groups relative to the P and S groups. The SP group in particular showed no cases of adhesive failure and an increased TBS value, due to strong chemical bonding. This demonstrates that surface treatments that target chemical bond strength contribute considerably more to the bond strength between zirconia and enamel than do surface treatments that enhance micro-mechanical bond strength. In addition, it suggests that, if applied in the clinic, the surface treatment method for the SP group could be convenient and even more effective than the other surface treatment groups.

## 5. Conclusions

This study aimed to investigate the effects of different surface treatment methods on the surface characteristics and tensile bond strength of zirconia when bonded with enamel. The following conclusions are supported by our findings:

1. Starting with the lowest, the order of TBS values for different zirconia surface treatments was the P group, the S group, the SS group, and the SP group.
2. An increase in the TBS value of the treated surface was accompanied by a concomitantly higher rate of cohesive failure at the bonded surface between the treated zirconia and enamel.
3. Compared to the P group, the roughness of zirconia increased significantly after surface treatments (*p* < 0.05); moreover, the contact angle decreased significantly (*p* < 0.05) while the wettability increased, which indicated a strong relationship between surface roughness and wettability.
4. The surface treatment method of applying MDP-containing silane primer after 110 µm Al_2_O_3_ sandblasting (SP group) is clinically convenient and effective at providing considerable improvements in the chemical bond strength between the zirconia and enamel.

## Figures and Tables

**Table 1:** Groups of 3Y-TZP (3 mol % yttria-stabilized tetragonal zirconia polycrystal) surface treatments performed in this study.

Groups (*n* = 15)	Surface Treatments
P	Polishing
S	110 µm A1_2_O_3_ Sandblasting
SS	110 µm A1_2_O_3_ Sandblasting + Selective Infiltration Etching (SIE) (i) Glass coating; (ii) Heating at 900 °C for 1 min; (iii) Cooling at 750 °C for 1 min; (iv) Re-heating at 900 °C for 20 min; (v) Cooling at room temperature; (vi) 5% HF etching for 30 min in an ultrasonic bath; (vii) Washing with distilled water for 5 min in an ultrasonic bath
SP	110 µm A1_2_O_3_ Sandblasting + MDP-containing silane primer
